# Anesthetic Implications of Factor XI Deficiency: A Clinical Case Study and Review of Literature

**DOI:** 10.7759/cureus.72594

**Published:** 2024-10-28

**Authors:** Pedro-Rafael Martinez-Lopez, Alejandro Barroso-Gonzalez

**Affiliations:** 1 Anesthesiology and Reanimation, Hospital Regional Universitario of Málaga, Malaga, ESP

**Keywords:** blood component therapy, hemoderivate management, hemophilia c, obstetric anesthesia, point-of-care viscoelastic testing

## Abstract

Factor XI deficiency, a rare but significant coagulopathy, poses unique challenges in perioperative management, particularly in obstetric settings. This review provides an in-depth exploration of the pathophysiology, diagnosis, and anesthetic implications of factor XI deficiency, thereby emphasizing the useful role of anesthesiologists. The variable bleeding phenotype of the disorder necessitates a nuanced understanding and tailored management strategies to mitigate severe perioperative bleeding risks. Conventional coagulation tests, while useful, often fall short in predicting bleeding risks, underscoring the importance of advanced diagnostic tools, such as viscoelastic testing. Viscoelastic testing provides real-time data on clot stability, which allows for immediate intervention and more targeted therapeutic strategies compared to standard coagulation tests. A clinical case of a 25-year-old patient with factor XI deficiency undergoing emergency surgery for an ectopic pregnancy illustrates the application of viscoelastic testing in managing acute bleeding and optimizing patient outcomes and advocates for the development of standardized protocols, continuous monitoring techniques, and enhanced training programs to improve the perioperative care of patients with factor XI deficiency, providing anesthesiologists with the tools necessary to navigate the complexities of factor XI deficiency in the perioperative environment. Integrating these advanced diagnostic and therapeutic approaches could significantly improve patient safety and surgical outcomes in patients with complex coagulopathy.

## Introduction

Factor XI deficiency, characterized by inadequate production of coagulation factor XI, leads to a range of bleeding severity, often triggered by surgery or trauma, significantly complicating anesthetic management during surgical interventions. Factor XI, a plasma glycoprotein that is part of the intrinsic pathway of blood coagulation, plays a relevant role in the amplification of coagulation. Deficiencies in factor XI can result in inadequate thrombin generation and impaired fibrin clot formation, which are critical processes during surgical and other invasive procedures. However, the clinical presentation of factor XI deficiency can vary widely between affected individuals, making the management of these patients particularly challenging. Some patients may remain asymptomatic and undiagnosed until they are exposed to hemostatic challenges, such as surgery, trauma, or obstetric procedures, where they may experience unexpected severe bleeding [1,2].

Traditionally, the diagnosis of factor XI deficiency is based on the activated partial thromboplastin time test (aPTT), which can be prolonged. However, aPTT may not always correlate with the risk of bleeding, which requires the use of additional diagnostic tools [3]. Recent advances in diagnostic methodologies, particularly the use of viscoelastic testing, such as thromboelastography (TEG), rotational thromboelastometry (ROTEM), and sonorheometry (Quantra), have provided new insights into the real-time coagulation status of patients [4]. These tests are instrumental in evaluating the dynamic properties of clot formation and degradation, offering a more nuanced understanding of the hemostatic challenges specific to factor XI deficiency [5,6]. Viscoelastic testing has also been pivotal in guiding the administration of targeted therapies during the perioperative period, potentially reducing the risk of bleeding and thrombotic complications [4,7].

The treatment strategies for managing factor XI deficiency during surgical procedures have evolved considerably. The use of fresh frozen plasma (FFP) and factor XI concentrates, when available, has been the cornerstone of treatment to prevent and control bleeding [8]. However, these treatments are not without risks, including volume overload and the potential transmission of infectious agents. More recently, antifibrinolytic agents, such as tranexamic acid, have shown promise in reducing perioperative bleeding without the risks associated with plasma components [9]. The role of recombinant factor replacement therapy is also being explored, which offers a safer and more effective means of treating bleeding in these patients [10,11].

This article thoroughly examines the anesthetic implications of factor XI deficiency, beginning with a clinical case that highlights real-world management challenges. Then it explores the epidemiology and genetics of the condition, discusses its pathophysiology, and reviews diagnostic strategies such as viscoelastic testing. Finally, it details perioperative management, which has been published as a therapeutic approach, focusing on anesthetic techniques, hemostatic therapies, and the importance of multidisciplinary collaboration.

## Case presentation

The theoretical concepts and management strategies discussed are further illustrated through the following clinical case, which underscores the practical challenges and solutions in managing a patient with factor XI deficiency undergoing an emergency surgical procedure.

Patient profile

A 25-year-old woman with no significant medical history except for a known factor XI deficiency (40%) presented to the emergency department with a diagnosis of five-week ectopic pregnancy. She had severe vaginal bleeding and a drop in hemoglobin level of 1 g/dL in a three-hour window, indicating significant blood loss. Despite initial hemodynamic stability, the patient showed signs of progressive hypotension and tachycardia, raising concerns about possible hemorrhagic shock due to her hematologic disorder.

Initial evaluation and preoperative management

**Figure 1:**
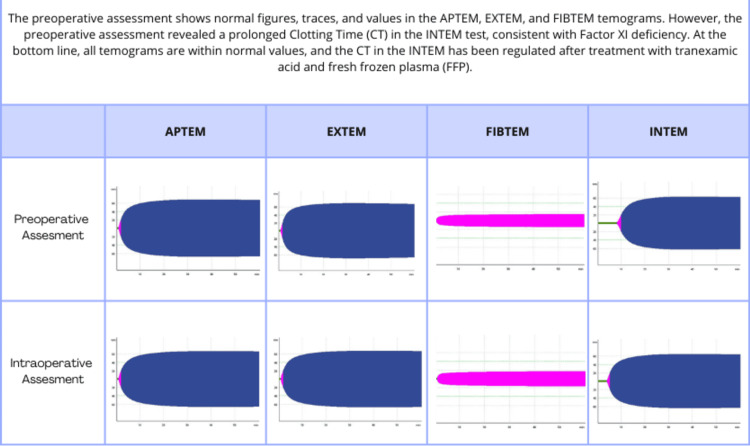
Rotational thromboelastometry (ROTEM) traces of the clinical case APTEM: anti-fibrinolysis; EXTEM: extrinsic coagulation activity assay; FIBTEM: fibrinogen level; INTEM: ellagic acid activated intrinsic pathway

Considering the patient’s factor XI deficiency and the nature of her bleeding, it was relevant to stabilize her coagulation profile before proceeding with surgery. The decision was made to administer 1 g of tranexamic acid and 250 ml of FFP preoperatively. Viscoelastic testing, particularly ROTEM, was performed prior to treatment to fully assess coagulation status (Figure 1).

Treatment and adverse reactions

In the anesthesia preparation room, following the administration of tranexamic acid and FFP, the patient developed a transfusion reaction characterized by heat, flushing, sweating, nausea, and general discomfort. This reaction was accompanied by a worsening of the hemodynamic instability. Immediate administration of corticosteroids resulted in resolution of these symptoms and stabilization of the patient’s hemodynamic status. Although the transfusion reaction posed risks, the decision to proceed was based on the patient’s deteriorating condition and the necessity to manage her ectopic pregnancy promptly.

Surgical intervention

**Figure 2:**
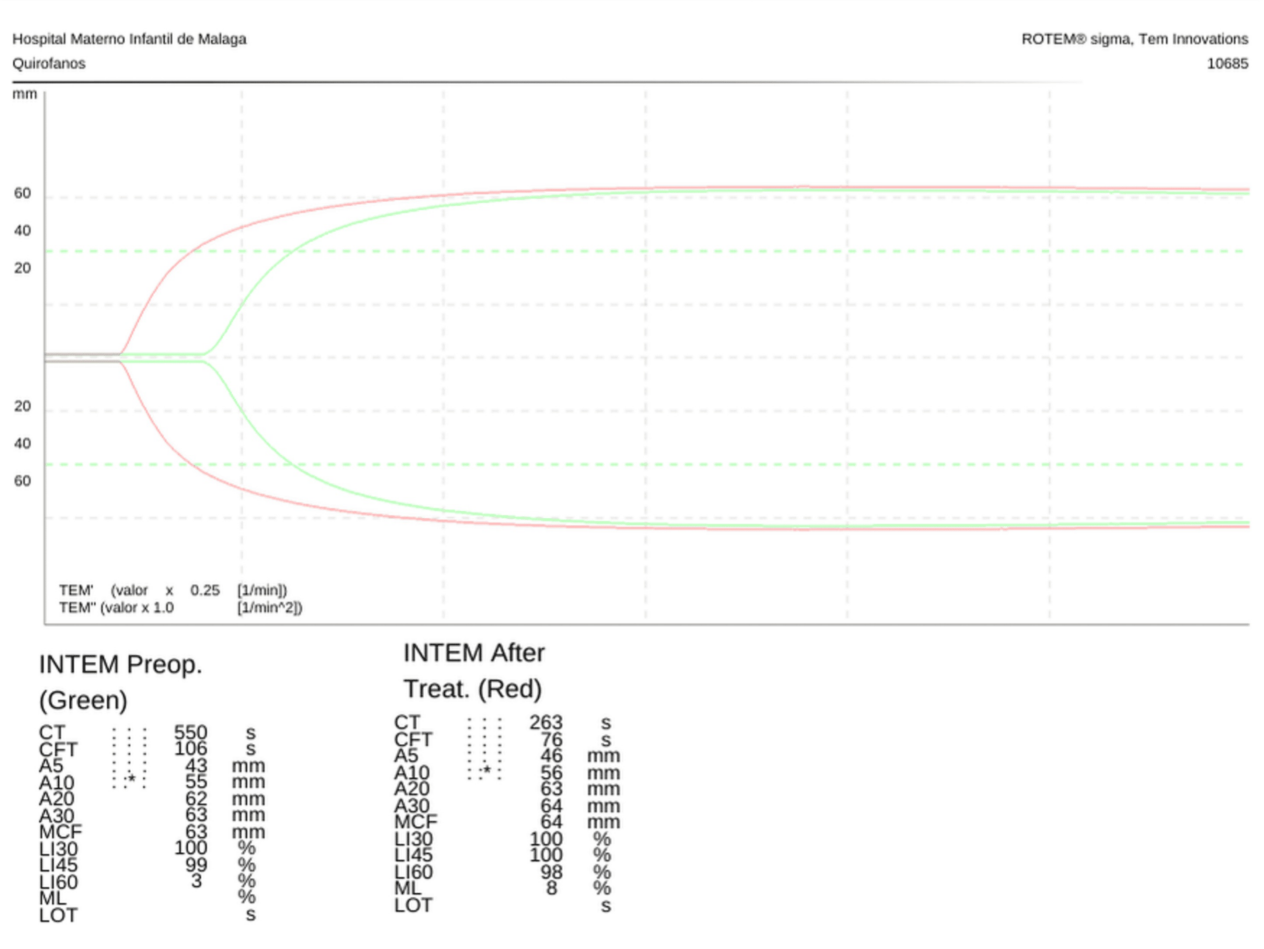
Rotational thromboelastometry (ROTEM) ellagic acid activated intrinsic pathway (INTEM) traces of the clinical case

After the resolution of the transfusion reaction, the patient underwent laparoscopic surgery for ectopic pregnancy. A second viscoelastic test performed preoperatively indicated that the coagulation abnormalities had been corrected with the administered treatment, as evidenced by the normalized clotting parameters. This correction provided greater assurance that the patient would not experience significant bleeding during surgery (Figures 1, 2). Consistent with the advanced diagnostic capabilities of viscoelastic testing outlined in the diagnostic review section, the results of the ROTEM provided actionable real-time data that informed our targeted preoperative interventions, significantly mitigating the risk of perioperative bleeding.

**Figure 3:**
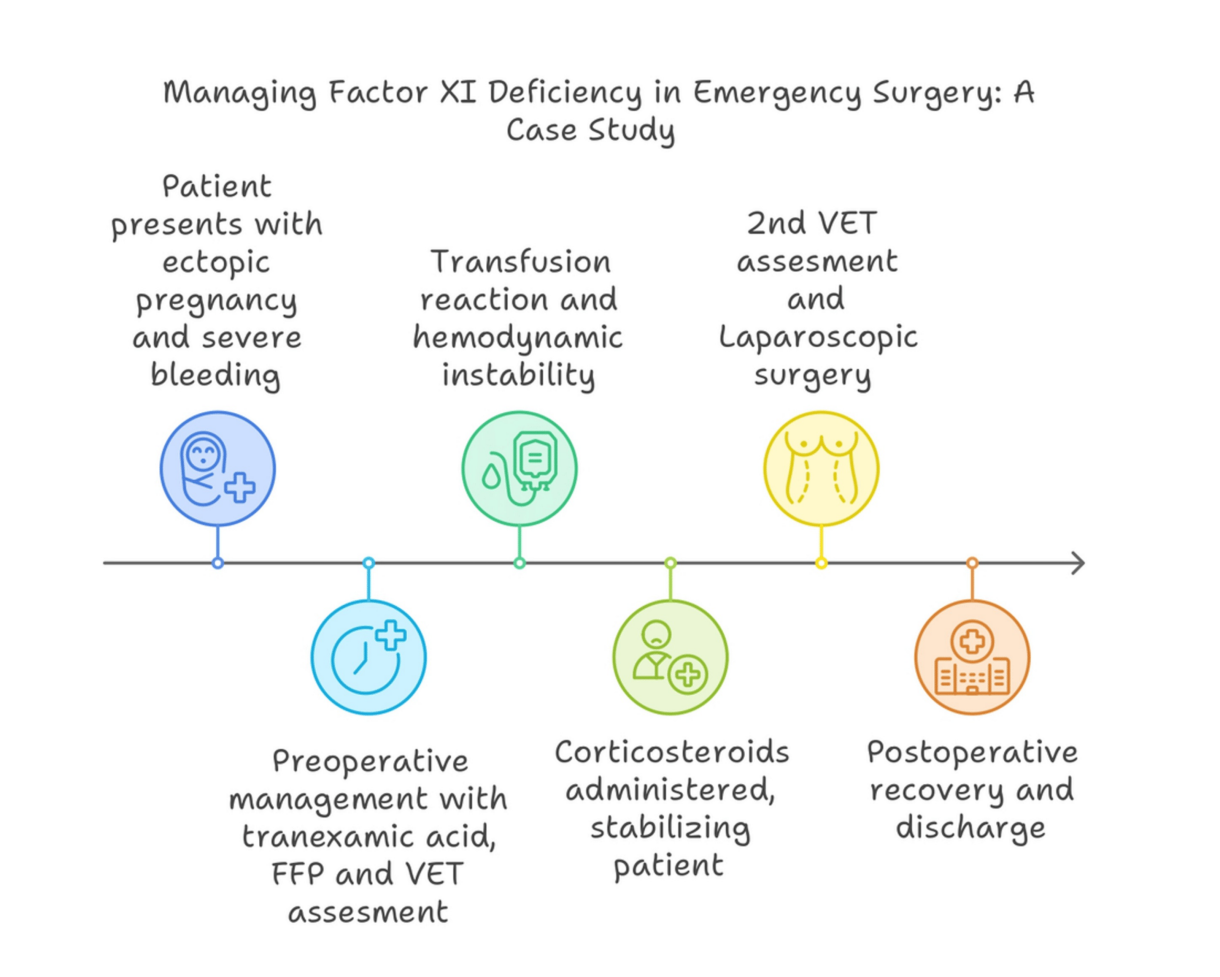
Managing factor XI deficiency in emergency surgery VET: viscoelastic testing; FFP: fresh frozen plasma

Surgery was performed without bleeding. Anesthesia was maintained without complications, demonstrating the efficacy of preoperative treatment in stabilizing the patient’s coagulation profile. As highlighted in the treatment strategies section, tranexamic acid and FFP are pivotal in managing factor XI deficiency. In this case, their administration, despite resulting in a transfusion reaction, was crucial for stabilizing the patient's coagulation profile ahead of surgery (Figure 3).

Postoperative care

Postoperatively, the patient remained hemodynamically stable and was transferred to the post-anesthesia care unit (PACU) for monitoring. She remained there for several hours with optimal recovery and showed no signs of bleeding or further complications. After 36 hours of observation and stable recovery in the ward, the patient was discharged home.

This case exemplifies the challenges and complexities of managing factor XI deficiency in a critical setting. Reinforcing the need for standardized protocols, advanced diagnostic tools, and comprehensive training programs discussed in our review's future directions, highlighting how clinical practice could evolve with ongoing research and education.

## Discussion

Epidemiology and genetics of factor XI deficit: prevalence, geographical distribution, and genetic bases

Factor XI deficiency is a rare autosomal bleeding disorder. Its epidemiology reveals significant variations in prevalence across different populations, with a particularly high heterozygote frequency of approximately 9% among the Ashkenazi Jew population, contrasting sharply with its rarity in other groups, where severe deficiency is often as low as one in 1,000,000 in the White population. The extensive genetic heterogeneity of this condition is evidenced by more than 190 mutations identified in the F11 gene, each contributing differently to different ethnicities. In addition, available evidence suggests that some individuals with inherited factor XI deficiency could develop inhibitors (antibodies directed against factor XI) if they were exposed to exogenous factor XI from factor XI concentrates or plasma. The risk of inhibitors appears to be especially increased with nonsense mutations such as the F11 Glu135stop allele [12].

The geographical distribution of factor XI deficiency further underscores its variable incidence. For instance, the mutation types prevalent among Ashkenazi Jews are rarely found in other populations, suggesting a strong founder effect and genetic drift in this group. In contrast, specific mutations are observed at variable frequencies in other ethnicities such as Africans, East Asians, and Latino populations, reflecting broader genetic variability and the impact of population-specific evolutionary histories [13].

The mutation spectrum in factor XI deficiency is not only broad but also complex, involving various mutation types that affect the gene in different ways, including missense, nonsense, and frameshift mutations. This complexity is reflected in the clinical presentation of the disorder, where the same mutation can result in different bleeding risks among individuals, highlighting the importance of personalized genetic screening and management approaches to effectively address the challenges posed by this coagulopathy in different ethnic and demographic settings. Tailored approaches appear to be essential to effectively mitigate the risk of bleeding during surgical or obstetric procedures, as well as to ensure that therapeutic interventions are optimally aligned with each patient’s unique hemostatic profile.

Furthermore, there are other causes of reduced factor XI levels, such as acquired factor XI inhibitors (auto- and alloantibodies). For instance, cancer or autoimmune diseases such as systemic lupus erythematosus may be associated with factor XI autoantibodies. These inhibitors may be detected in the evaluation of unexplained bleeding or during the management of an individual with known factor XI deficiency when FFP or factor XI concentrates fail to raise the factor XI level or if the bleeding pattern worsens. Other acquired conditions may also lead to reduced factor XI levels along with reduced levels of other coagulation factors, such as acute disseminated intravascular coagulation or liver disease.

Pathophysiology of factor XI deficiency

Factor XI deficiency poses unique challenges due to its variable bleeding phenotype and potential for severe perioperative bleeding. Factor XI functions as a critical component of the intrinsic coagulation pathway, wherein it is primarily responsible for amplifying the generation of thrombin, a key enzyme in fibrin clot formation [10]. Factor XI is activated through two primary mechanisms: by factor XIIa in the presence of high molecular weight kininogen and negatively charged surfaces, or more pivotally by thrombin in a feedback loop that sustains and amplifies clot formation.

Factor XI deficiency leads to a bleeding disorder that is most apparent during surgical procedures or injuries, particularly in tissues with high fibrinolytic activity. This is because factor XI not only contributes to thrombin generation but also plays a significant role in stabilizing the fibrin clot against premature lysis by affecting the activation of thrombin-activatable fibrinolysis inhibitor, which inhibits fibrin degradation. Therefore, a deficiency in factor XI disrupts this balance, leading to an increased risk of bleeding.

Recent studies in animal models have further elucidated the role of factor XI in thrombosis, demonstrating that while factor XI deficiency affects thrombus formation, it surprisingly does not lead to excessive bleeding, suggesting a therapeutic potential for targeting factor XI to prevent thrombotic disorders without significant bleeding risks. On the other hand, some scientific studies have suggested that the rates of cardiovascular events and venous thromboembolism appear to be reduced in individuals with factor XI deficiency [14]. These observations have generated enthusiasm about the therapeutic reduction of factor XI levels as a form of thromboprophylaxis.

Clinically, the risk of bleeding associated with factor XI deficiency is notoriously difficult to predict due to the nonlinear relationship between factor XI levels and the severity of bleeding. Individuals with a severe deficiency may not exhibit bleeding symptoms unless challenged by surgery or trauma. This unpredictable nature of the bleeding phenotype complicates management strategies and necessitates individualized treatment plans for the affected patients. Understanding the complex pathophysiology of factor XI deficiency lays the groundwork for the development of sophisticated diagnostic techniques, such as viscoelastic testing.

Review of the diagnosis and monitoring coordination in factor XI deficiency: integrating conventional techniques and viscoelastic testing

Diagnosis and management of factor XI deficiency, particularly in the perioperative setting, require specific attention to both conventional and specialized diagnostic methods. 

Differential Diagnosis

The differential diagnosis of factor XI deficiency included other bleeding disorders: First, hemophilia A and B are congenital bleeding disorders due to deficiencies in factor VIII and factor IX, respectively. There is often a positive family history of aPTT, and both cause isolated prolonged aPTT. These conditions can be easily distinguished by determining their factor levels. Secondly, Von Willebrand disease is an inherited bleeding disorder due to a deficiency of the Von Willebrand factor. The aPTT test may be normal in some individuals with mild Von Willebrand disease. Unlike factor XI deficiency, this disease has normal factor XI levels. Thirdly, acquired factor inhibitors (autoantibodies) can be developed against coagulation factors. Inhibitors that affect the intrinsic pathway can cause bleeding and prolonged aPTT. The activities of the factors involved are low. Lastly, antiphospholipid antibodies are autoantibodies directed against phospholipid-protein complexes; they can cause the lupus anticoagulant phenomenon, with a prolonged clotting time (typically aPTT) in vitro, but do not cause clinical bleeding. However, these individuals may be at risk of thrombosis if they have antiphospholipid syndrome.

Conventional Coagulation Monitoring

Conventional coagulation assays, such as prothrombin time (PT) and aPTT, are fundamental in the diagnosis of factor XI deficiency. These tests, particularly aPTT, are sensitive to factor XI levels and are routinely used to confirm the deficiency [5]. However, their limitations include the following: On the one hand, these tests have limited predictive value for bleeding risk; although useful for diagnosis, aPTT does not correlate well with the risk of bleeding in patients with factor XI deficiency. In the same way, aPTT is not useful for managing any patient undergoing procedural and surgical interventions beyond using it as a guide for anticoagulation therapy using heparin or direct thrombin inhibitors.

On the other hand, the delayed turnaround is remarkable; the results from these tests often have a long turnaround time, which can delay decision-making in acute settings.

To address these limitations, more specialized tests, including assays for specific factor XI activity, are used to confirm the diagnosis and gauge the severity of the deficiency. Although obtaining factor XI levels is a useful tool, it is not available in many centers, and reliance on aPTT for screening remains standard.

Genetic Screening

Genetic testing is not required for the diagnosis of factor XI deficiency. In addition, given that genetic identity is not consistently related to phenotypic disease, genetic screening for factor XI is not routinely performed in the general population because this would be an unnecessary expense. Therefore, it may be only beneficial in selected cases, such as individuals of Ashkenazi Jewish descent, because if the patient is injured or undergoes surgery, it would allow the patient to consider prophylactic therapies as necessary. However, screening all Ashkenazi Jewish women with genetic testing might prove impossible with little yield and false confidence in the results, so we also should review the patient’s medical history focus on any previous bleeding episodes, and perform it in each case after consulting a geneticist.

The Role of Viscoelastic Testing in Diagnosis and Treatment

Viscoelastic testing has become relevant in the personalized treatment of bleeding disorders during surgery in numerous hospitals [7]. These tests are especially valuable in surgical and critical care settings for patients with coagulation disorders such as factor XI deficiency due to their ability to measure various aspects of clot formation and stability in real time, providing immediate insights relevant to managing acute bleeding episodes, which allows guided blood component use and treatment monitoring, reducing the risk of fluid overload and transfusion-related complications.

Perioperative management protocols for factor XI deficit: anesthetic strategies

Factor XI deficiency presents distinct challenges in perioperative management, especially in obstetric settings, where both patient and fetal outcomes depend significantly on optimal anesthetic care [2]. Detailed planning is required to effectively manage the heightened risk of bleeding. Therefore, this risk necessitates a comprehensive approach to perioperative care that begins with a thorough preoperative evaluation to determine the patient’s bleeding history and current factor XI levels [10].

Preoperative Preparation

Literature published suggests that effective management should start with a thorough preoperative assessment that includes reviewing the patient’s medical history and focusing on any previous bleeding episodes or surgical interventions. In addition, aPTT should be obtained before procedures. Regarding chronic treatment, we may advise people with factor XI deficiency to avoid non-steroidal anti-inflammatory drugs and other antiplatelet agents due to concerns about an increased risk of bleeding, unless there is a cardiovascular indication. 

First of all, it is important to consider the chronic anticoagulation or antiplatelet therapy. Decisions concerning anticoagulation or antiplatelet therapy must be individualized based on the bleeding phenotype and the consequences of withholding antithrombotic therapy. Anticoagulation studies suggest that standard-dose anticoagulation can be used in individuals with factor XI deficiency if needed, although only a small number have been evaluated. Evidence suggests that dual antiplatelet therapy should be used with great caution, although data are limited. Low-dose aspirin as a single agent appears to be tolerated in most patients [15].

Secondly, we should know factor XI levels. Based on available scientific evidence, factor XI levels should be quantitatively assessed in patients with known deficiency, as they do not always correlate directly with bleeding risk; some patients with very low levels may not bleed excessively, while others with higher levels might. This variability makes preoperative planning critical. Despite its importance, obtaining levels of factor XI is available depending on the geographic location. Fortunately, more and more centers count on this useful tool at present.

Thirdly, we may check the availability of blood components. Ensuring the availability of appropriate blood components, such as FFP or factor XI concentrates, appears to be relevant. These should be ready to be used if needed to manage acute bleeding episodes during surgery.

Moreover, it is essential for multidisciplinary team coordination. Collaboration with the hematology, obstetric, and anesthesia teams is relevant to developing a personalized management plan that addresses both the risks of bleeding and thrombosis, including advance planning for elective surgery. 

Hemostatic Therapies

On the one hand, it is important to consider antifibrinolytic agents. The use of an antifibrinolytic agent has been published as effective in reducing bleeding risk and in treating bleeding, especially at sites of fibrinolytic activity (nasopharynx area, genitourinary tract, and endometrium). Tranexamic acid is available in doses of 1 g four times a day (orally, 15-25 mg/kg every six to eight hours; or intravenously, 10 mg/kg every six to eight hours). Therapy could be started approximately two to 12 hours before surgery and continued for approximately seven days. However, caution is recommended when using tranexamic acid alongside factor XI concentrate, as it may increase the risk of thrombotic events in patients with elevated factor XI levels [10].

On the other hand, replacement products of factor XI (FFP and concentrate factor XI) are a useful option. According to the FFP, the amount of factor XI in plasma is equivalent to 1 unit/mL, so FFP administered at a dose of 10-20 mL/kg has been proven to be usually sufficient to raise the factor XI level to 10%-20% above the baseline level. The main safety concerns associated with FFP are volume overload, acute transfusion-related lung injury, allergic reactions, and transfusion-transmitted infections [8]. Pathogen-inactivated products, such as solvent-/detergent-treated plasma, have the theoretical advantage of reducing exposure to enveloped viruses without any increase in adverse events. However, these products are expensive.

With reference to the factor XI concentrate, a dose of approximately 15 units/kg is suggested by scientific evidence as usually sufficient to increase the factor XI level to >30 percent. Purified factor XI concentrates have been reported to cause thrombotic complications (arterial and venous) in up to 10% of individuals, according to studies [16]. Therefore, patients at a high risk of bleeding should undergo an evaluation of the specific risks of the procedure, and it may be appropriate to use a lower dose of factor XI concentrate or an alternative source of factor XI, such as FFP. Factor XI is not present in cryoprecipitate or prothrombin complex concentrates (PCCs), so these products have not demonstrated value in the treatment of isolated factor XI deficiency.

In addition, we may take into account the recombinant activated factor VII in inhibitor development. Individuals at high risk of inhibitor development (antibodies) may choose to use a bypassing agent such as recombinant activated factor VII rather than factor XI replacement in some settings, such as bleeding or surgery, to reduce the risk of developing an inhibitor. Based on the available literature, a typical dose of activated factor VII recombinant used is 15 to 30 mcg/kg intravenously, especially in individuals with severe factor XI deficiency [17]. 

Moreover, desmopressin is a possible option. Subcutaneous or intravenous administration of desmopressin could prevent clinical bleeding when used for surgery in heterozygous individuals with factor XI deficiency, based on evidence. The typical dose of desmopressin is 0.3 mcg/kg.

Intraoperative Management

The choice of anesthetic technique, whether regional or general, should be carefully considered based on the individual’s bleeding risk assessed during preoperative preparation, in addition to employing meticulous surgical techniques to minimize tissue trauma and using local hemostatic agents when necessary to significantly reduce intraoperative bleeding. This may include topical products such as fibrin and sealants.

According to the management of acute bleeding, serious acute bleeding should be treated with factor XI replacement products to increase the factor XI activity level from 30% to 45%. Supported by researchers, co-administration of an antifibrinolytic agent is often suggested as appropriate for severe bleeding, as this may improve hemostasis in areas of high fibrinolytic activity with a low risk of adverse events. Minor bleeding can be managed with an antifibrinolytic agent alone, especially in areas with high fibrinolysis [18].

Regarding severe bleeding in a patient with a factor XI inhibitor, researchers support that individuals with inhibitors (based on the failure of the factor XI level to increase as expected with factor XI replacement) may use recombinant activated factor VII [19]. Often, coadministration of an antifibrinolytic agent appears to be appropriate, as this may improve hemostasis in areas of high fibrinolytic activity with a low risk of adverse events.

Role of Viscoelastic Testing in Management

Scientific evidence suggests that viscoelastic testing can be used to immediately identify coagulation abnormalities, allowing timely intervention with appropriate blood components or coagulation factors [20].

First of all, viscoelastic testing provides real-time monitoring and adjustment. The real-time monitoring capacity of the viscoelastic testing allows immediate adjustments in therapeutic interventions, which is particularly beneficial during surgery. By providing real-time data, viscoelastic testing allows for adjustments in therapy during procedures, potentially reducing unnecessary transfusions and improving medical management. 

Secondly, viscoelastic testing allows guided therapy and reduced transfusion. The ability of viscoelastic testing to provide a comprehensive assessment of clot stability and fibrinolysis helps guide the selective use of blood components, such as FFP and platelets, thus minimizing the risk of fluid overload and transfusion-related complications.

Thirdly, we can avoid transfusion reactions using viscoelastic testing. The occurrence of transfusion reactions in our patients emphasizes the need for preparedness and rapid response strategies. This underscores the importance of having protocols in place for the treatment of transfusion reactions, particularly in patients with known coagulation disorders who are at increased risk for such complications. This case highlights the necessity of balancing the benefits of transfusion of blood components with the risks, ensuring that patients receive only what is necessary to correct their coagulation profile without causing adverse reactions.

Discussion

The pathophysiology of factor XI deficiency further complicates perioperative management due to its role in the intrinsic pathway of blood coagulation. Factor XI deficiency leads to inadequate thrombin generation and impaired clot stability, particularly in tissues with high fibrinolytic activity, such as the uterus, during pregnancy [2]. This could result in severe bleeding during surgical procedures, highlighting the need for meticulous perioperative planning and management [11,21,22]. Therefore, the treatment of factor XI deficiency in the perioperative setting, particularly in obstetric patients, presents unique challenges that underscore the important role of anesthesiologists. This role includes preoperative assessment, intraoperative management, and postoperative care [2,8], all of which are important for minimizing bleeding risks and ensuring patient safety.

Several guidelines and recommendations have been proposed for the management of patients with factor XI deficiency. For instance, published literature suggests recommendations for the management of patients with factor XI deficiency during pregnancy [23]. However, according to the role of hemostatic therapies in factor XI deficiency, such as antifibrinolytic agents and factor XI replacement products, there are several unresolved issues in previous publications. In that way, providing a more comprehensive overview of the appropriate use of antifibrinolytic agents in the management of bleeding as well as their doses appears to be a primary objective. Moreover, it seems important to incorporate detailed considerations of prophylactic measures, including the use of factor concentrates, lysine analogs, and low to moderate doses of plasma.

Importance of the Anesthesiologist

The clinical case discussed provides a clear illustration of the complexities involved and the importance of comprehensive diagnostic and therapeutic strategies, together with the anesthesiologist’s expertise, which is valuable in developing a personalized management plan that addresses the specific risks associated with factor XI deficiency. In this case, the use of viscoelastic testing was crucial for real-time monitoring, allowing for rapid adjustments to hemostatic management that minimized the risk of bleeding during surgery [22] (Figures 1, 2). The initial test provided real-time detailed insights into the patient’s coagulation status, allowing for targeted preoperative interventions, such as the administration of tranexamic acid and FFP. The ability of the viscoelastic testing to provide immediate feedback on the patient’s coagulation status allowed rapid adjustments to the treatment plan, significantly reducing the risk of perioperative bleeding [22].

On the other hand, knowing factor XI levels is important for an anesthesiologist to carefully adapt intraoperative management. A retrospective multicentre observational study was conducted by Flaujac et al. in French hemostasis centers on pregnant women with factor XI deficiency. The results supported the use of a 30 IU/dL factor XI level threshold for neuraxial anesthesia (all types of neuraxial anesthesia in women with no bleeding history [24]: spinal and epidural) at the time of the procedure or for catheter removal, despite normal factor XI activity ranges between 60 and 150 IU/dL in a healthy parturient in the third trimester. However, they also reported a 17.5% incidence of postpartum hemorrhage or excessive postpartum bleeding, in accordance with previous studies [24].

Utilization of Viscoelastic Tests

The incorporation of viscoelastic testing in management algorithms has been proposed by researchers to refine the perioperative treatment of factor XI deficiency [25]. Scala et al. developed an algorithm based on viscoelastic testing sigma parameters that effectively tailored interventions for coagulopathic bleeding, underscoring the diagnostic and therapeutic relevance of these tests in complex coagulopathies like factor XI deficiency [25].

Supported by Research

At this point, the research underscores the benefits of integrating viscoelastic testing in the management of Factor XI deficiency. Papers such as those by Beneš et al. and by Ganter and Hofer highlight the efficacy of viscoelastic testing in various clinical settings, demonstrating its utility in guiding hemostatic therapy and improving patient management [22,26].

Rotational thromboelastometry, in particular, is highlighted in evidence published for its utility in assessing the intrinsic pathway of coagulation, which is directly affected by factor XI activity [6]. Recent studies have emphasized the pivotal role of this assessment in the perioperative management of patients with factor XI deficiency. For instance, viscoelastic testing assays have been successfully used to guide the administration of FFP and other blood components during surgery, thereby significantly optimizing patient outcomes and minimizing unnecessary transfusions. For instance, a case reported by Kazui et al. demonstrated that viscoelastic testing-guided FFP administration could effectively manage a patient with severe factor XI deficiency undergoing cardiac surgery, avoiding excessive transfusion and its associated risks [27].

Comparison with Other Hematologic Disorders

The use of viscoelastic testing extends beyond factor XI deficiency and has shown similar benefits in other hematologic conditions, such as hemophilia and Von Willebrand disease [28], based on available scientific evidence. For instance, in hemophilia, viscoelastic testing has been utilized to monitor the effectiveness of factor replacement therapy and guide the administration of antifibrinolytic agents [29,30]. Similarly, in Von Willebrand disease, viscoelastic testing has proven useful in assessing the overall hemostatic potential and tailoring treatment regimens to patient-specific needs.

In conclusion, factor XI deficiency is a complex disorder with significant implications for hemostasis and thrombosis. Understanding its pathophysiology not only aids in better clinical management but also opens avenues for research into more effective treatments that target the nuanced roles of factor XI in the coagulation and fibrinolytic systems. 

Areas for improvement and future directions

Firstly, it is essential to the development of protocols. The literature available suggests that there is a clear need to develop standardized protocols for the perioperative treatment of patients with factor XI deficiency. These protocols should encompass guidelines for viscoelastic testing, management of transfusion reactions [31], and optimal use of antifibrinolytic agents and factor XI concentrates. Protocols should also address the management of possible transfusion reactions and the judicious use of blood components to minimize risks.

Secondly, we should enhance monitoring techniques. According to researchers, continuous viscoelastic testing monitoring could further improve the treatment of coagulopathy in surgical settings by providing ongoing real-time data and allowing immediate adjustments in therapy. 

Thirdly, viscoelastic testing leads to improvements in training and education. Continued education and training programs for healthcare providers on the use of viscoelastic testing and the management of transfusion reactions could be useful. This ensures that all team members are prepared to respond effectively to complications based on data support. 

Lastly, viscoelastic testing allows better hemoderivative management in obstetric care. Management of blood components is of paramount importance in obstetric care. Establishing protocols for the use of hemoderivatives could help reduce unnecessary transfusions and minimize the risk of transfusion reactions. Scientific evidence suggests that the precise administration of blood components, guided by viscoelastic testing, ensures that patients receive only what is necessary, thus improving results and conserving valuable resources. 

## Conclusions

Factor XI deficiency presents unique and significant challenges in the perioperative management of patients, particularly in obstetric settings. This discussion of the literature based on a clinical case report highlights the critical role of the anesthesiologist in ensuring patient safety and optimizing surgical outcomes. According to researchers, the development and implementation of standardized protocols, continuous monitoring techniques, and comprehensive training programs could be useful for improving the management of factor XI deficiency. By adopting these advanced diagnostic and therapeutic approaches, healthcare providers can enhance patient outcomes and ensure safer surgical experiences for those with complex coagulopathies. The integration of advanced diagnostic tools such as viscoelastic testing appears to be significant for real-time monitoring and adjustment of therapeutic interventions, based on available evidence. These tests can provide detailed information on the patient’s coagulation status, allowing for targeted and effective management strategies that minimize the risk of bleeding and avoid unnecessary transfusions. 
